# Metaphase I orientation of Robertsonian trivalents in the water-hyacinth grasshopper, *Cornops aquaticum* (Acrididae, Orthoptera)

**DOI:** 10.1590/S1415-47572009005000019

**Published:** 2009-01-23

**Authors:** Pablo César Colombo

**Affiliations:** Departamento de Ecología, Genética y Evolución, Facultad de Ciencias Exactas y Naturales, Universidad de Buenos Aires, Ciudad Universitaria, Buenos AiresArgentina

**Keywords:** * Cornops aquaticum*, Robertsonian rearrangements, trivalents, metaphase I orientation

## Abstract

Trivalents resulting from polymorphic Robertsonian rearrangements must have a regular orientation in metaphase I if the polymorphisms are to be maintained. It has been argued that redistribution of proximal and interstitial chiasmata to more distal positions is necessary for a convergent orientation, the only one that produces viable gametes. *Cornops aquaticum* is a South-American grasshopper that lives and feeds on water-hyacinths, and has three polymorphic Robertsonian rearrangements in its southernmost distribution area in Central Argentina and Uruguay. The orientation of trivalents in metaphase I, the formation of abnormal spermatids and the frequency and position of chiasmata in the trivalents, was analysed in a polymorphic population of *C. aquaticu*s. In this study we observed a correlation between the number of trivalents with the frequency of abnormal spermatids; additionally, the number of chiasmata, especially proximal and interstitial ones, was strongly correlated with the frequency of the linear orientation. Therefore we confirmed our previous assumption, based on other evidence, that the chiasmata redistribution in fusion carriers is essential to the maintenance of the polymorphisms.

Robertsonian translocations (also called centric fusions) are frequently involved in the evolutionary divergence of plants and animals (Capanna, 1982; Baker and Bickham, 1986; King, 1993). When hybrids are formed between species or chromosomal races differing in one or several Robertsonian rearrangements the behaviour of the resulting trivalent(s) is frequently erratic, leading to linear configurations in metaphase I that cause the formation of imbalanced gametes. These heterozygotes for Robertsonian rearrangements present negative heterosis, as their fertility is reduced due to the non-disjunctional orientation in metaphase I. Robertsonian rearrangements were thus included in models of chromosomal speciation, as negative heterosis is a necessary step in these models (White, 1978; King, 1993). No polymorphism can be maintained with negative heterosis on fitness (Hedrick, 1983), but Robertsonian rearrangements are frequently polymorphic (Bidau, 1990; Colombo, 1989; Fan and Fox, 1991; Mayr *et al.*, 1984; Narain and Fredga, 1998; Pascoe *et al.*, 1996, to mention only a few). In these cases, the depressing effects of structural heterozygosis on the fitness of heterozygotes are suppressed. In fact, in most studied cases of polymorphic centric fusions, trivalents showed convergent orientation, linear orientation being the exception to the rule (Bidau and Mirol, 1988; Mirol and Bidau, 1991, 1992).

How do trivalents manage to succeed? One possibility is preadaptation. Robertsonian translocations between similar sized acrocentrics are more likely to do well in metaphase I. Likewise, distal chiasmata increase the probability of convergent orientation, given that too many proximal and/or interstitial chiasmata would spatially hinder the bending of the trivalent at the centromere. However, if chiasma formation is unrestricted, Robertsonian rearrangements could trigger proximal and interstitial chiasmata redistribution to more distal positions. This is actually a frequent feature of centric fusions (Bidau, 1990; Colombo, 1989, 1990, 1993, 2007; Davison and Akeson, 1993; Dumas and Britton-Davidian, 2002; Hewitt & Schroeter, 1968; see Colombo, 2007, for a discussion).

The water-hyacinth grasshopper *Cornops aquaticum* Bruner is a New World acridid that lives, feeds and oviposits on plants of the genus *Eichhornia* (Adis and Junk 2003, Adis *et al.* 2004). It has been considered for release in Africa as a natural control of water-hyacinths (Oberholzer and Hill 2001), which have become a serious water weed (Centre *et al.*, 2002). *C. aquaticum* natural distribution is between the south of Mexico and east-central Argentina and Uruguay (between 23° N and 35° S). Three polymorphic Robertsonian rearrangements that follow a North-South cline were found in the southernmost extreme of *C*. *aquaticum* geographic distribution (Mesa, 1956; Mesa *et al.*, 1982; Colombo, 2008). The chromosomes involved in these centric fusions have their chiasma distribution severely affected, shifting from proximal and interstitial to more distal positions (Colombo, 2007). In this work, we analysed the relationship between proximal and interstitial chiasma frequency and linear orientation. We also studied the correlation between non-convergent orientation and the formation of abnormal spermatids.

Twenty-one out of the 27 males used in this study were from two highly polymorphic populations of *Cornops aquaticum* from Zárate and Baradero, Argentina, on the Paraná River, during the Austral Summers of 2005, 2006 and 2007. Other three males from a mostly monomorphic population of *C. aquaticum* (Tigre, close to Buenos Aires) were used and represented the “zero trivalent” class. The males were dissected and their testes were fixed in 3:1 ethanol:acetic acid. Cytological preparations were made by squashing some follicles of the testis in propionic haematoxylin. Metaphase I plates were scored for trivalent chiasma frequency and position and for trivalent orientation (linear or convergent). In order to classify chiasma positioning, the arms of the trivalents were divided in three. Chiasmata located in the proximal third of the chromosome arm were called “proximal” (P), those located in the intermediate third were classified as “interstitial” (I) and those present in the distal third were called “distal” (D). Signs of structural tension were taken into account when analysing trivalent orientation. If no tension was observed, the cell was considered to be in prometaphase I and was not computed. This consideration is important because the inclusion of these cells in the analysis would lead to an overestimation of linear orientation. In order to assess abnormal spermatids, we counted the number of “centriolar adjuncts” (the root of the future flagellum) establish whether a spermatid was haploid, diploid or polyploid. Statistical tests were performed with the STATISTICA package (StatSoftInc, Tulsa OK, USA).

Trivalent orientation can be either convergent or linear (Figures 1A and B). In each of the males we observed if there was any relationship between the frequency of linear orientation and the number and position of chiasmata (Table 1). Normality of the variable “percentage of linear orientation” was assessed by means of a Kolmogorov-Smirnov test which was not significant (p = 0.09788), thus not ruling out normality. Total chiasma frequency among linearly and among convergently oriented trivalents was compared by means of an ANOVA and differences were highly significant (p = 0.000374). In order to check if chiasma position was relevant for metaphase I orientation, we performed a MANOVA with proximal, interstitial and distal chiasma frequencies as variables and orientation (linear or convergent) as the grouping factor. The differences were highly significant (p = 0.001). As for univariate effects, differences were highly significant for proximal chiasmata (p = 0.001134) as well as for interstitial ones (p = 0.008164), whereas differences were significant for distal chiasmata (p = 0.024743). When proximal chiasmata were taken into account, the means were 0.4123 for linearly oriented trivalents and 0.1093 for convergently oriented ones. For interstitial chiasmata, the means were 0.7140 for linear trivalents and 0.3565 for convergent ones; and for distal chiasmata, means were 1.5388 for linear trivalents and 1.7247 for convergent ones. It is therefore clear that a high number of chiasmata favours linear orientation (Figures 1A, B and solid arrows). Among these, proximal and interstitial chiasmata mostly precluded convergent orientation, whereas distal chiasmata were associated with convergent orientation.

Individuals with one, two and three trivalents and without heterozygous fusions were compared with respect to the formation of diploid or tetraploid spermatids (Table 2). The variable “proportion of abnormal spermatids” did not show a normal distribution. The Kolmogorov-Smirnov test rendered non-significant values (p = 0.1024319) when the variable was transformed to log10, thus showing normal distribution. The regression of “proportion of abnormal spermatids (transformed)” on “number of trivalents” was significant (p = 0.019919). The regression of “percentage of abnormal spermatids (transformed)” on “percentage of linear orientation” was not significant (p = 0.591304).

*Cornops aquaticum* is not a favourable material for the study of aneuploidy in metaphase II, since cells in these meiotic stage are scarce and the plates are of bad quality (Figures 1C and D). Only five out of 149 metaphase II plates analysed presented aneuploidy. Anaphase I cells were also rare but their quality was much better. A total of 53 anaphase I cells were analysed and the segregation of trivalents was always normal, with the submetacentric moving towards one pole and the two corresponding acrocentrics migrating towards the other.

Centric fusions are usually accompanied by extensive chiasma repatterning and shifting of chiasma position in acrocentric bivalents to more distal positions in Robertsonian bivalents or trivalents (Bidau, 1990; Colombo, 1989, 1990, 1993, 2007; Davison and Akeson, 1993; Dumas and Britton-Davidian, 2002; Hewitt and Schroeter, 1968). However, the cause for chiasma redistribution in Robertsonian bivalents may not be the same as in Robertsonian trivalents. When chromosome races are compared, the race with the lowest diploid number frequently has less (proximal) chiasmata than the race with a higher diploid number (Davison and Akeson, 1993; Dumas and Britton-Davidian, 2002). We attribute this difference to interference across the centromere. In fact, ever since Mathers work (1938), the centromere has been considered a barrier to the operation of interference. Nevertheless, Colombo and Jones (1997) demonstrated that interference does operate across the centromere in the grasshopper species *Leptysma argentina* and *Chorthippus brunneus*. The same was demonstrated in humans with an improved statistical method (Broman and Weber, 2000).

Another factor must operate in trivalents. When there is a trivalent, the synaptonemal complex (SC) is interrupted at the level of the centromere. It has been frequently suggested that either the operation of interference needs a complete SC (Sym and Roeder, 1994) or that SC formation needs the action of interference. Anyway, there is consensus that both SC formation and interference are related. It thus follows that interference should not operate across the centromere in trivalents.

It must be pointed out that in trivalents, it is always *polymorphic*, and never polytypic or spontaneous centric fusions that present chiasma redistribution to more distal positions. Indeed, chiasma frequency and position are usually unchanged in spontaneous centric fusions (Colombo, 1987; López-Fernández *et al.*, 1984; Sannomiya, 1968; Southern, 1967). Concurrently, when studied, more than 36% of metaphase I plates showed linear orientation. (Colombo, 1987; Kayano and Nakamura, 1960; López-Fernández *et al.*, 1984; Teoh and Yong, 1983). Bidau *et al.* (2001) studied chiasma frequency and distribution in a hybrid zone of *Mus musculus.* In this case, the individuals studied were natural hybrids between a race with four fixed Robertsonian rearrangements and another one with all acrocentric chromosomes. The race with four submetacentric bivalents showed a decreased number of proximal and interstitial chiasmata, as expected, but hybrids showed trivalents with *increased* proximal and interstitial chiasma frequencies. The orientation of trivalents in metaphase I was not studied.

We thus conclude that chiasma repatterning towards more distal positions in the case of Robertsonian trivalents is restricted to polymorphic conditions. The suppression of proximal and interstitial chiasmata is clearly adaptive for the maintenance of the polymorphism, given that proximal and interstital chiasmata are correlated with the frequency of linear orientation and thus with the origin of aneuploid and/or polyploid gametes (Bidau and Mirol, 1988, Mirol and Bidau, 1991, 1992). In this study, the correlation between the proportion of abnormal spermatids and the percentage of linear orientation was not significant. However, we were unable to correlate the formation of *aneuploid* spermatids and the linear orientation because we only identified *polyploid* spermatids. Nevertheless, we can assume that linear orientation leads to the formation of aneuploid sperm, even if in some cases reorientation may occur.

Whether or not the reorganisation of chiasma frequency and position was triggered by natural selection favouring the maintenance of the polymorphism or by the rearrangement itself is largely a matter of speculation. If the latter is true, only preadapted centric fusions (*i.e.*, those that cause chiasma repatterning) would survive in a polymorphic state. In either case, the fact that no abnormal anaphase I and very few aneuploid metaphases II had been detected reflects the stability of the polymorphisms herein studied.

Lagging chromosomes or trivalents may result in polyploid spermatids (John *et al.*, 1983). We thus studied polyploid spermatids in order to evaluate the degree of infertility caused by cytological heterozygosis. This approach can only detect the infertility caused by *polyploid* spermatids and that due to *aneuploid* spermatids should be studied with more sophisticated techniques (Lowe *et al.*, 1996). The analysis revealed a correlation between the percentage of polyploid spermatids and the number of trivalents (Figure 2), thus confirming the relationship between these two variables. This result coincides with those found by Bidau and Mirol (1988) in *Dichroplus pratensis*. The percentage of polyploid spermatids was extremely low, corroborating the stability of the polymorphisms herein studied.

**Figure 1:**
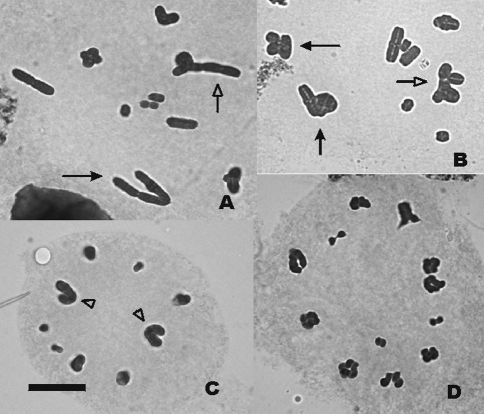
Metaphase plates of *Cornops aquaticum* showing orientation and segregation of the trivalents*.* (A) Metaphase I with two trivalents, one with convergent orientation (solid arrow) and the other with linear orientation (hollow arrow); (B) Metaphase I with three trivalents, two with convergent orientation (solid arrows) and one with linear orientation (hollow arrow); (C) Metaphase II with two submetacentric (arrowheads) and seven acrocentric chromosomes; (D) Metaphase II with 12 acrocentric chromosomes. Bar = 10 μm.

**Figure 2:**
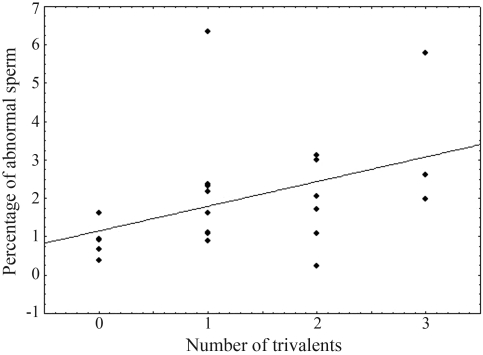
Regression analysis of the “percentage of abnormal spermatids” in relation to the number of trivalents *per* individual.

## Figures and Tables

**Table 1:** Metaphase I orientation of trivalents and chiasma frequency *in Cornops aquaticum.*

	N	L	P	I	D	P'	I'	D'
B06002	34	5.88	0	1.50	1.00	0.06	0.31	1.66
B06003	186	0.54	0	0	2.00	0.04	0.04	1.93
B06001	91	5.49	1.20	0.20	1.80	0.13	0.21	1.81
Z06001	140	7.14	0.30	0.20	1.80	0.04	0.07	1.93
Z06002	138	2.90	0.50	1.00	1.25	0.04	0.44	1.60
Z06005	123	2.44	0	0.67	2.00	0.03	0.14	1.96
Z06004	175	0.57	0	2.00	1.00	0.37	0.59	1.81
Z07001	274	2.92	0.87	0.75	1.25	0.07	0.48	1.63
Z07002	178	2.25	0.25	0.50	1.50	0.06	0.04	1.87
Z07007	93	5.38	0.80	0.10	1.20	0.19	0.41	1.83
Z07004	106	3.77	0	0.25	1.75	0.01	0.03	2.00
Z07005	148	0.68	0.27	0.88	1.75	0.24	0.84	1.57
Z07008	236	4.66	0.63	0.82	1.36	0.12	0.49	1.42
Z06013	74	8.11	0.33	1.50	1.50	0.06	0.91	1.69
Z05027	204	4.90	0.60	0.20	1.40	0.11	0.20	1.80
Z05024	379	2.64	0	0.60	1.80	0.06	0.11	1.94
Z05005	284	13.73	0.13	0.67	1.33	0.08	0.35	1.79
Z05020	101	4.95	0.63	0.40	1.60	0.29	0.78	1.46
Z05004	254	3.15	0.63	0.22	1.13	0.19	0.56	1.40
Z05012	58	8.62	1.20	1.20	1.60	0.08	0.70	1.35
Z05010	83	7.23	0.33	0.50	2.00	0.03	0.19	1.78

N = Total number of cells; L = percentage of linear orientation; P, I, D = proximal, interstitial and distal frequency of chiasmata in trivalents with linear orientation; P', I' D' = proximal, interstitial and distal frequency of chiasmata in trivalents with convergent orientation.

**Table 2:** Metaphase I orientation of trivalents in *Cornops aquaticum* and formation of abnormal spermatids.

	n	L	S	A	N
B06002	34	5.88	nd	nd	1
B06003	186	0.54	1583	1.08	2
B06001	91	5.49	986	2.60	3
Z06001	140	7.14	1962	1.08	1
Z06002	138	2.90	1059	2.32	1
Z06005	123	2.44	787	6.35	1
Z06004	175	0.57	1389	1.61	1
Z07001	274	2.92	1136	1.97	3
Z07002	178	2.25	2191	0.23	2
Z07007	93	5.38	1196	1.10	1
Z07004	106	3.77	756	3.00	2
Z07005	148	0.68	1093	2.05	2
Z07008	236	4.66	1310	1.71	2
Z06013	74	8.11	nd	nd	2
Z05027	204	4.90	nd	nd	2
Z05024	379	2.64	nd	nd	2
Z05005	284	13.73	661	3.12	2
Z05020	101	4.95	476	5.78	3
Z05004	254	3.15	1387	2.36	3
Z05012	58	8.62	2116	2.17	1
Z05010	83	7.23	1896	0.90	1
Z06003	-	-	967	1.63	0
Z06007	-	-	962	0.93	0
Z06006	-	-	1623	0.67	0
Ti06017	-	-	1198	0.92	0
Ti06015	-	-	1553	0.39	0
Ti06016	-	-	1235	0.64	0

Total	3359		21984		

N = Total number of cells; L = percentage of linear orientation; S = total number of recorded spermatids; A = percentage of abnormal spermatids; N = number of trivalents.
